# MicroRNA-133a and MicroRNA-145 May Be Involved in the Development of Hypertension-Mediated Organ Damage in Children with Primary Hypertension—A Preliminary Study

**DOI:** 10.3390/jcm13226929

**Published:** 2024-11-18

**Authors:** Michał Szyszka, Piotr Skrzypczyk

**Affiliations:** 1 Department of Pediatrics and Nephrology, Doctoral School, Medical University of Warsaw, 02-091 Warsaw, Poland; mszyszka@wum.edu.pl; 2 Department of Pediatrics and Nephrology, Medical University of Warsaw, 02-091 Warsaw, Poland

**Keywords:** microRNA, primary hypertension, adolescents, hypertension-mediated organ damage

## Abstract

**Background/Objectives:** Studies in adults have demonstrated the essential role of microRNAs in developing hypertension and their effect on hypertension sequelae. In this preliminary study, we aimed to investigate the expression of five miRNA particles, miRNA-21, miRNA-27a, miRNA-27b, miRNA-133a, and miRNA-145, in school-aged children with primary hypertension and to examine their correlations with blood pressure and arterial and heart properties. **Methods:** In 22 hypertensive children (15.1 ± 1.9 years), we measured blood pressure parameters (office, central, and 24 h), the urinary albumin/creatinine ratio, and the pulse wave velocity (PWV) before and after one hour of aerobic exercise. The left ventricular mass index (LVMI) and common carotid artery intima–media thickness (cIMT) were also assessed. The relative miRNA expression was calculated using the 2^−ΔΔCt^ method with miRNA-16 as an endogenous control and the pre-exercise miRNA expression levels as the control (baseline). **Results:** We found a statistically significant decrease in both the office and 24 h ambulatory diastolic blood pressure after 1 h of exercise (82.2 ± 8.5 mm Hg versus 78.6 ± 8.8 mm Hg, *p* = 0.01 and 75.0 ± 8.3 mm Hg versus 73.0 ± 7.4 mm Hg, *p* = 0.02). The increase in miRNA-133a expression after exercise correlated positively with the LVMI. Furthermore, the rise in miRNA-145 expression after exercise correlated negatively with the systolic and diastolic office and 24 h blood pressure and with markers of arterial damage: 24 h PWV and cIMT. **Conclusions:** In conclusion, miRNA-133a may be a biomarker of left ventricular hypertrophy in children with elevated blood pressure. Additionally, changes in miRNA-145 expression induced by exercise might reduce the blood pressure after exercise and protect against arterial damage. Both miRNA-133a and miRNA-145 may be involved in epigenetic alterations in children affected by primary hypertension that may contribute to the exacerbation of HMOD.

## 1. Introduction

MicroRNAs (miRNAs) are single-stranded, non-coding, evolutionarily conserved RNA molecules that regulate post-transcriptional gene expression in all eukaryotic cells [1]. It has been more than two decades since the discovery of the first two microRNA molecules, named lin-4 and let-7, in the nematode *C. elegans*. The latter was also found as the first known human miRNA. The discovery of these unique molecules was even awarded this year’s Nobel Prize in Physiology or Medicine (2024); Victor Ambros and Gary Ruvkun were recognized for their discovery and for demonstrating their role in post-transcriptional gene regulation. In recent years, there has been growing interest in these peculiar molecules, which have been studied extensively in oncology, particularly for their emerging therapeutic applications. However, studies also point to the multiple effects of miRNAs on processes in cardiovascular diseases, including hypertension, as well as diabetes or kidney diseases [2]. There are promising results when looking at the adult population but there are no data available for pediatric patients with primary hypertension. This is particularly important given the significant contribution of genetic factors to the development of primary hypertension, which is much greater in children and is not masked by as many environmental factors that accumulate over a lifetime.

Arterial hypertension (AH) is found in approximately 4% of pediatric patients, with a growing prevalence with age [3]. From puberty, AH is 3–4 times more frequent in boys than in girls. Primary hypertension (PH) is related to half of all cases of pediatric patients with high blood pressure and is the most common form of arterial hypertension in adolescents. The vast majority of primary hypertension cases are asymptomatic, and the first manifestations show up when organ damage, such as heart failure, myocardial infarction, stroke, atherosclerosis, renal failure, or vision impairment, has already developed. Arterial hypertension was the leading global contributor to premature death in 2015, accounting for almost 10 million deaths. Most of the deaths were due to ischemic heart disease (4.9 million), hemorrhagic stroke (2.0 million), and ischemic stroke (1.5 million) [4].

PH is a complex and multifactorial disease, and several mechanisms are involved in its development. Genetic and environmental factors, the sympathetic nervous system, the hormonal–enzymatic renin–angiotensin–aldosterone system, and substances produced by the endothelium—nitric oxide and endothelins—play an essential role in this process. Genes play quite a significant role in BP regulation as well. A parental history of AH increases the risk of the disease in offspring, especially if both parents are hypertensive. Epidemiological studies have suggested that in arterial hypertension, 60% of cases have a genetic background (association), and 40% are environmental [5]. However, the exact process of hypertension development is not fully explained, and molecular pathways still have unexplored gaps.

Regular physical activity is an essential aspect of one’s lifestyle and is used as a non-pharmacological strategy for preventing and treating early-stage hypertension or as an adjunct to pharmacological intervention in more advanced cases. The American Heart Association recommends at least 30 min of aerobic exercise at least five days a week to promote cardiovascular risk reduction [6]. It is well established that acute and chronic aerobic exercise lowers blood pressure in adults. An immediate reduction in BP after a dynamic exercise session has been termed PEH (post-exercise hypotension); the effect is seen up to 24 h after the end of the exercise. Scientific data suggest that the reduction in BP is more significant in patients not on antihypertensive medications [7]. There are less data on this topic in the pediatric population. However, preliminary evidence suggests that aerobic training can reduce BP and significantly reverse endothelial damage also in this group [8]. A study in obese children showed that regular exercise can lower BP by as much as seven mmHg in 3 months [9]. This effect is independent of reducing body fat. As for microRNA, it has been found that the expression of various miRNAs was up- or downregulated in healthy volunteers up to 72 h after a single session of exercise [10]. Furthermore, some miRNAs show the opposite form of expression in hypertension and after exercise. There is a lack of data on the relationship between these miRNA particles and the occurrence of indicators of hypertension-mediated organ damage (HMOD), and none of these particles have been studied in the pediatric population.

Our preliminary project aimed to check the expression of five microRNA particles (miRNA-21, miRNA-27a, miRNA-27b, miRNA-133a, and miRNA-145) in pediatric patients with PH. After analyzing the available literature in this field, we selected the above five particles, which might be involved in the development of arterial hypertension and HMOD in children and adolescents.

## 2. Materials and Methods

### 2.1. Study Group

As a preliminary study, the aim was to establish a proper protocol before recruiting a larger group of patients for a more thorough investigation. All 22 patients were recruited for this single-center observational study from individuals with primary hypertension who presented to the ward and outpatient clinic of our university hospital without previous treatment with antihypertensive drugs.

The inclusion criteria were as follows: not yet pharmacologically treated arterial hypertension diagnosed following up-to-date recommendations of the European Society of Hypertension [11] and confirmed in ambulatory blood pressure monitoring, height ≥ 120 cm, and signed informed consent to participate in the study.

Patients with secondary forms of hypertension, severe kidney, liver, or heart disease that could influence the study’s results, or any inflammatory disease, e.g., acute infection at the time of the study (temporary exclusion criterion), were excluded from the study.

This preliminary study analyzed the first consecutive 22 patients who qualified for the final study.

### 2.2. Basic Clinical Parameters

Upon admission, all patients underwent an assessment of basic anthropometric parameters, including height (in centimeters), weight (in kilograms), and body mass index (BMI) (in kilograms per square meter). We compared these anthropometric measurements with Polish normative data and expressed them as Z-scores [12]. According to the World Health Organization definition, children with BMI Z-scores > 1 and >2 were considered overweight and obese, respectively [13].

A series of interviews were conducted to gather information on the subjects’ birth weight, gestational age, and family history of hypertension.

### 2.3. Carrying Out Aerobic Training

To ensure the same exercise load for each study patient, an ergospirometric test was performed before the standardized exercise session to determine the maximal oxygen uptake (VO_2_ max) for each participant (using METALYZER 3B ergospirometry device during the ramp exercise test with the protocol for children, Cortex Biophysics GMBH, Leipzig, Germany). Then, during the study, all participants performed 60 min of aerobic exercise on the CYCLUS 2 ergometer (RMB elektronik-automation GMBH, Leipzig, Germany), corresponding to 65% of their VO_2_ max (steady-state cycling exercise), under the supervision of the investigator (MS). Patients were instructed to maintain their usual physical activity and diet and not to eat 2 h before exercise. After the training session, they continued with their daily routine.

### 2.4. Parameters of Blood Pressure and Hypertension-Mediated Organ Damage

The methodology employed for BP measurement and the assessment of arterial damage was described in detail in our previous manuscripts [14,15,16,17]. In brief, peripheral office BP was evaluated oscillometrically by the Omron HBP-1320 (OMRON HEALTHCARE, Co., Ltd., Kyoto, Japan) (mmHg) and Z-scores [12]. The Mobil-O-Graph device with 24 h pulse wave analysis and pulse wave velocity (I.E.M. Industrielle Entwicklung Medizintechnik GmbH, Stolberg, Germany) was used to conduct 24 h ambulatory blood pressure monitoring (ABPM). The data were interpreted following the recommendations for pediatric patients outlined in the cited article [18]. The following parameters were included in the final analysis: systolic, diastolic, and mean arterial blood pressure (SBP, DBP, MAP, respectively, mmHg), as well as systolic and diastolic central blood pressure (cSBP, cDBP, respectively, mmHg). We also analyzed the following hemodynamic parameters: the augmentation index normalized to the heart rate of 75 beats per minute (AIx75HR) (%), total vascular resistance (TVR) (dyn·s/cm^5^), stroke volume (SV) (mL), and pulse wave velocity (PWV) analysis conducted over 24 h. Furthermore, 24 h heart rate (beats per minute) and nocturnal blood pressure dip (%) were measured. We also evaluated carotid–femoral pulse wave velocity using applanation tonometry (the Sphygmocor device [AtCor Medical Pty Ltd., Sydney, Australia]). The Aloka Prosound Alpha 6 (Hitachi Aloka Medical, Mitaka, Japan), equipped with a 13 MHz linear transducer, was employed to measure common carotid artery intima–media thickness (cIMT) (mm). The cfPWV and cIMT were presented as numeric values and Z-scores, as detailed in the following references [19,20].

In addition to the assessment of artery-associated HMOD (cIMT, atPWV), the albumin-to-creatinine ratio (ACR) (mg/g) in the first-morning portion of urine and the hypertrophy of the left ventricle expressed as the left ventricular mass index (LVMI) (g/m^2^, 7 or g/m^2^) were also evaluated. The ACR was measured by the hospital laboratory using standard laboratory techniques. An ACR greater than 30 mg/g was considered an indicator of renal damage caused by hypertension [11].

Echocardiography was conducted using the Philips iE33 xMATRIX (Echocardiography System, Philips Healthcare, Amsterdam, The Netherlands) and a wide-spectrum probe with frequency adjusted to the patient’s body mass index. Following the guidelines set forth by the European Society of Hypertension and the Polish Society of Hypertension, the LVMI was indexed for height^2.7^ in children aged ≤ 15. Left ventricular hypertrophy (LVH) was identified if the left ventricular mass index (LVMI) was equal to or exceeded the 95th percentile for age and gender [21]. In adolescents aged ≥ 16 years, left ventricular mass was indexed for body surface area, calculated using the DuBois and DuBois formula. The body surface area (BSA) is calculated as follows: BSA = 0.20247 × height (m)^0.725^ × weight (kg)^0.425^. Left ventricular mass index (LVMI) is defined as LVM/BSA. In adolescents aged ≥ 16 years, left ventricular hypertrophy will be identified if the left ventricular mass index (LVMI) is >115 g/m^2^ for boys and >95 g/m^2^ for girls [21].

### 2.5. Additional Biochemical Parameters

In all patients, serum creatinine (mg/dL), uric acid (mg/dL), serum sodium (mmol/L) and potassium (mmol/L), total, low-density lipoprotein (LDL), high-density lipoprotein (HDL) cholesterol (mg/dL), and triglycerides (mg/dL), were measured using standard laboratory procedures employed by the hospital department. Normal values of lipid parameters were taken from Stewart et al. [22]. In all the participants, we calculated the glomerular filtration rate (GFR) from the revised creatinine-based Schwartz formula in children under 16 and the CKD-EPI in those who were equal to or above the age of 16 [23].

### 2.6. Evaluation of miRNA

Blood for miRNA analysis was collected using a vacuum technique in K2-EDTA tubes (BD Vacutainer^®^, Beckton, Dickinson and Company, Franklin Lakes, NJ, US). Each blood sample was centrifuged within 10 min at 3000× *g* for 15 min at 4 °C, and the supernatant was centrifuged again under the same conditions to remove any trace of blood cells. In addition, a visual inspection was performed to detect hemolysis. Hemolyzed samples were excluded from further analysis. Plasma was transferred to cryotubes and frozen at −80 °C until laboratory analysis. To protect against the degradation of microRNAs, the plasma samples were handled only with extended, sterile, double-filtered pipette tips free of RNase, DNase, PCR inhibitors, endotoxin, and DNA. There was no additional freeze–thaw cycle; all samples were used only once. miRNA extraction was performed using commercially available mirVana PARIS kits (Thermo Fisher Scientific, Waltham, MA, USA) according to the manufacturer’s instructions with modifications based on our experience. The isolated RNA’s quantity, quality, and purity were measured by spectrophotometry using the NanoDrop Lite (Thermo Fisher Scientific, Waltham, MA, USA). For reverse transcription, commercially available TaqMan kits (Thermo Fisher Scientific, Waltham, MA, USA) were used following the manufacturer’s protocol with modifications based on our experience. Real-time PCR was performed using commercially available TaqMan Advanced miRNA Assays (Thermo Fisher Scientific, Waltham, MA, USA) and the LightCycler 480 II (Roche Diagnostics GmbH, Mannheim, Germany). According to the available literature on plasma miRNA in PH, miRNA-16 expression was chosen as the normalization standard (endogenous control) [24], exogenous cel-miRNA-39 as isolation control, and exogenous cel-miRNA-54 as reverse transcription control. All samples were run in triplicate. Data were analyzed using the LightCycler 480 II Software 1.5, and relative miRNA levels were calculated using the 2^−ΔΔCt^ method. As there was no control group in this preliminary study, the baseline microRNA expression level before exercise was used as the patient-specific control. All miRNA sequences are listed in Supplementary Material Table S1 with the corresponding accessions derived from miRbase accessed on 15 March 2023 (https://www.mirbase.org/) for clarity and possible further analysis, including others to replicate and build on the published results.

### 2.7. Statistical Methods

The authors archived the obtained data in an anonymized form (Excel 365, Microsoft 365, Microsoft, Redmond, WA, USA) with password-protected user-level access. Statistical analysis was performed with Dell Statistica 13.0 PL software (TIBCO Software Inc., Palo Alto, CA, USA). The normality of numeric datasets was tested using the Shapiro–Wilk test.

All continuous variables were presented as mean ± standard deviation (SD) and interquartile range (IQ). Subsequently, the following tests were used, depending on the data distribution: Student’s *t*-test for dependent variables, Wilcoxon’s test, Spearman’s rank correlation, and Pearson’s correlation. Test results with a *p*-value < 0.05 were considered of statistical significance.

## 3. Results

### 3.1. Clinical and Biochemical Data of the Studied Children

The baseline clinical and biochemical parameters in the studied children are shown in Table 1. Most of the study participants were boys (82%), and their mean age was 15 years. There were two premature children in the study group. The vast majority of the patients with PH were overweight or obese, and all the studied patients had normal renal function. Elevated or borderline cholesterol was found in 23% and 9%, elevated or borderline LDL-cholesterol in 9% and 14%, lowered or borderline HDL-cholesterol in 23% and 9%, and elevated or borderline triglycerides in 27% and 27% of the participants, respectively. Hyperuricemia was found in half of all the patients (50%). All the patients had normal serum sodium and potassium concentrations.

### 3.2. Blood Pressure of the Studied Children

Table 2 shows the office peripheral, ambulatory peripheral, and central blood pressure before and after a single bout of aerobic exercise. All patients had elevated systolic or diastolic office and ambulatory blood pressure. Based on the ABPM results, 59% of the patients had isolated systolic hypertension, and 41% of the subjects had systolic–diastolic hypertension. Seven patients were dippers, ten were non-dippers, four were extreme dippers, and one was a reverse dipper.

After one hour of a standardized exercise, we observed no significant changes in the systolic blood pressure. Still, there was a significant decrease in the office and 24 h diastolic and 24 h central diastolic blood pressure. There were also significant reductions in the 24 h stroke volume and total vascular resistance. After 60 min of exercise, the children’s heart rate was significantly higher. The 24 h pulse wave velocity remained the same before and after the exercise, and we found no difference in the augmentation index.

### 3.3. Hypertension-Mediated Organ Damage of the Studied Children

**Figure 1:**
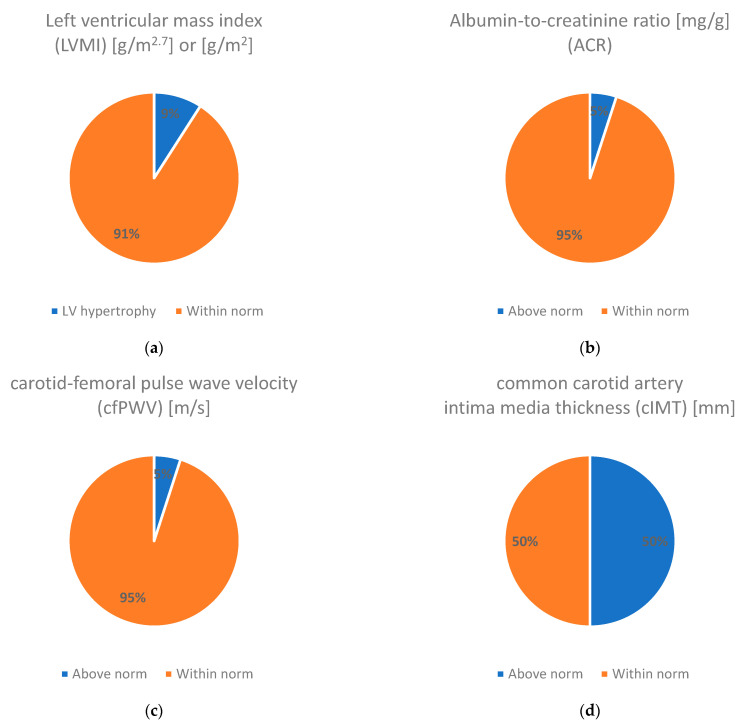
Hypertension-mediated organ damage indicators in the studied patients. (**a**) Left ventricular mass index ([g/m^2.7^] or [g/m^2^]) depending on age; (**b**) albumin-to-creatinine ratio (ACR) [mg/g]; (**c**) carotid–femoral pulse wave velocity (cfPWV) [m/s]; (**d**) common carotid artery intima–media thickness (cIMT) [mm].

The HMOD parameters are shown in Table 3 and Figure 1. We observed no significant differences between the ACR and cfPWV measurements before and after the standardized aerobic exercise sessions. Half of the patients had an abnormally thickened carotid intima–media. Two patients had left ventricular hypertrophy (LVH), one had elevated albuminuria, and one had abnormal cfPWV at the time of the study.

### 3.4. MicroRNA of the Studied Children

**Figure 2:**
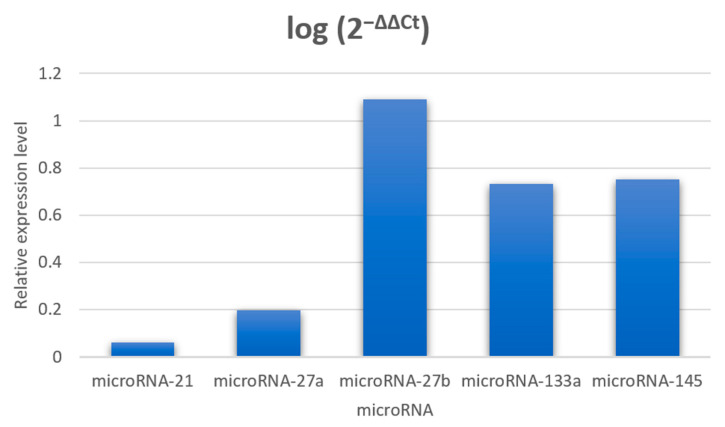
Relative microRNA expression levels in plasma (before and after exercise session) were calculated by the 2^−ΔΔCt^ method, with miRNA-16 expression level as endogenous control, and Ct before exercise as the norm.

**Figure 3:**
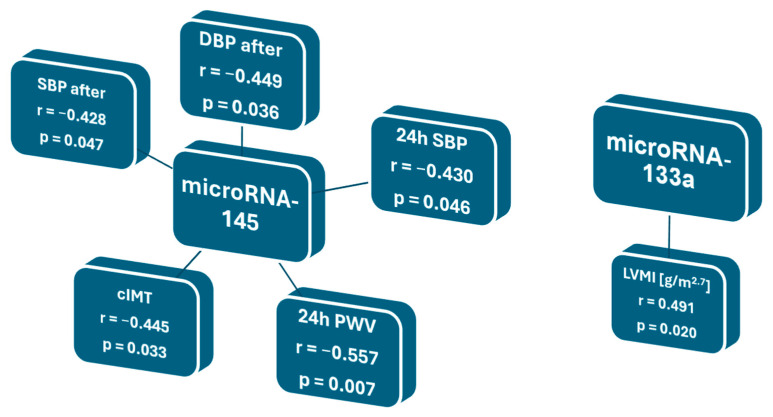
Significant correlations of relative microRNA expression levels in plasma with blood pressure parameters and hypertension-mediated organ damage indicators. SBP—systolic blood pressure, DBP—diastolic blood pressure, 24 h SBP—24 h systolic blood pressure measured by ambulatory blood pressure monitoring; cIMT—common carotid artery intima–media thickness, 24 h PWV—pulse wave velocity measured by 24 h ambulatory blood pressure monitoring device, LVMI—left ventricular mass index.

An increase in the relative expression level of all the microRNA molecules was observed but was not statistically significant for the study group (Figure 2). the molecules numbered 27b had the highest increase in expression, followed by those numbered 145 and 133a. The post-exercise increase in the microRNA-133a expression correlated positively with the LVMI [g/m^2.7^] (r = 0.491, *p* = 0.020). The post-exercise increase in the microRNA-145 expression levels correlated negatively with the post-exercise systolic and diastolic blood pressure, 24 h systolic blood pressure, 24 h peripheral PWV, and cIMT (r = −0.428, *p* = 0.047, r = −0.449, *p* = 0.036, r = −0.430; *p* = 0.046, r = −0.557, *p* = 0.007 and r = −0.455, *p* = 0.033, respectively) (Figure 3)—none of the tested microRNA molecules correlated with age.

## 4. Discussion

Our observational preliminary study analyzed the selected microRNAs as potential biomarkers of blood pressure parameters and subclinical arterial damage in twenty-two previously untreated children with primary hypertension. All of the microRNA particles were analyzed before and after a single 60 min standardized aerobic exercise. In addition, we evaluated the blood pressure parameters, the cfPWV, and the ACR before and after the exercise. We also looked at other indicators of hypertension-mediated organ damage, such as the LVMI and cIMT. Although the relative expression levels of the microRNAs measured did not increase significantly, all of them were overexpressed after the exercise session in this small group of patients. Interestingly, the study revealed significant correlations between microRNA-145 and blood pressure and arterial damage parameters. The microRNA-133a expression also correlated with the left ventricular mass index. The study also showed no difference between the PWV and ACR measured before and after the moderate-intensity exercise sessions and showed that the diastolic blood pressure decreased after exercise in both the office and 24 h measurements in the children with PH. After 60 min of exercise, the children’s heart rate was higher, and there were also significant reductions in the 24 h stroke volume and total vascular resistance.

Regular physical activity is a well-established, essential component of a healthy lifestyle. It is used as a non-pharmacological strategy for prevention and treatment in the early stages of arterial hypertension or as an adjunct to pharmacological intervention in more advanced cases. The American Heart Association recommends at least 30 min of aerobic exercise five days a week to promote cardiovascular risk reduction [6]. The BP-lowering effects of exercise are much more pronounced in people with arterial hypertension than in healthy people [7]. However, most of the data are from adults, including older people, and there is little evidence of this phenomenon in children. One randomized controlled trial showed a reduction in the systolic and diastolic blood pressure of 10 and 6 mmHg, respectively, after plyometric exercise [25]. Another study in obese children shows that BP can be reduced by as much as 7 mmHg in 3 months with regular exercise, and this effect is independent of body fat reduction [9]. In our study, the SBP was reduced by 4 mmHg and the DBP by 3 mmHg after a single bout of aerobic exercise in hypertensive children, supporting the above findings. Despite the small number of studied children, we found significant differences in the central and peripheral diastolic blood pressure. The diastolic blood pressure mainly depends on the arterial elasticity and peripheral (total vascular) resistance, which decreased in our group after exercise. The systolic blood pressure, on the other hand, depends on the work of the heart (stroke volume and heart rate) and also on the elasticity of the large arteries. There were opposite changes in the HR and SV after exercise in the children studied, which may explain the lack of a significant change in systolic pressure.

Studies in recent years have linked exercise to changes in microRNA expression. A recently published systematic review of the literature by Kotewitsch et al. identified microRNA molecules whose change in expression was replicated in multiple studies in adults. In response to resistance exercise, the expressions of microRNA-133a and microRNA-206 were altered in most of the analyzed studies. In response to endurance exercise, microRNAs 15a, 29c, 30a, 30e, 103a, 130a, 132, 142, 143, 155, 181a, 181b, 338, 451a, and let-7e showed consistent elevation in 100% of all the included studies, whereas microRNAs 103a, 130a, and let-7e were consistently downregulated [26]. The review’s authors linked the change in microRNA expression to the positive effects of exercise on elements of the innate and acquired immune system.

It also appears that, depending on the lifestyle and level of training, a single session of exercise may affect the expression of other microRNA molecules and thus have different clinical effects. Nair et al. showed that the profile of microRNAs altered by exercise in older adults is different in trained and sedentary individuals. This, in turn, may exert a different effect on the insulin-like growth factor 1 (IGF-1) signaling pathway and thus differently affect exercise-induced hypertrophy of skeletal muscle and cardiomyocytes [27]. Other studies, on the other hand, indicate that the dose of training may matter. Spanish authors have observed that different exercise doses induced specific microRNA profiles [28].

In many adult studies, the circulating levels of microRNA-133a were increased in healthy subjects after exercise training [29,30,31,32,33]. Interestingly, because miRNA-133a indirectly targets antihypertrophic genes including guanosine triphosphate-guanosine diphosphate (GDP-GTP) exchange protein, Rhoa, signal transduction kinase cell division control protein 42 (Cdc42), and the nuclear factor negative elongation factor complex member A (Nelfa/Whsc2), miRNA-133a downregulation is involved in the process of cardiac hypertrophy [34]. Our study suggests this effect is also present in hypertensive children, in whom microRNA-133a exhibited an increased relative expression after a training bout. However, its level was positively correlated with the LVMI. We hypothesize that children with LVH already have a greater potential to reverse unfavorable changes in the heart, and microRNA-133a is more abundant in these patients after exercise. However, further studies with a control group and more detailed studies of the molecular pathways are needed.

Interestingly, we found numerous negative correlations of changes in the microRNA-145 expression levels with blood pressure and the arterial damage parameters cIMT and PWV. Our study sheds new light on the role of this molecule in generating hypertension and organ changes. Our result somewhat contradicts the result of the study by Santovito et al. The authors showed significantly higher expression of microRNA-145 in atherosclerotic plaque collected during endarterectomy in patients with hypertension [35]. Moreover, experimental work suggests an adverse role for microRNA-145 in the development of both systemic and pulmonary hypertension [36,37]. In our group, microRNA-145 behaved differently, indicating its protective role. The expression of the particle increased after exercise and showed a negative correlation with pressure and organ changes. Some experimental data suggest that microRNA-145 might be a negative regulator of the expression of GATA binding protein 6 (GATA6), thus attenuating cardiomyocyte hypertrophy in the experimental model [38]. Other correlations in our work compared to other authors may have been due to different protocols for determining microRNA expression (including different centrifugation conditions and use of probes from various companies) and determination of microRNA in different materials (serum, plasma, cells, atherosclerotic plaques). Obviously, our pilot study cannot unequivocally assess the role of microRNA145 in the development of hypertension and its sequelae in the pediatric population (small patient group, no comparison with healthy children, no evaluation of expression in target organs). Studies on larger groups of patients, studies with a control group, and prospective studies would be needed. We hope that the final results of our project will provide insight into the role of this molecule.

In our small group, we further observed a post-exercise increase in the expression of other microRNA molecules: microRNA-27a, microRNA-27b, and microRNA 21. However, we did not show any significant correlation between the change in the expression of these molecules and blood pressure or hypertension-mediated organ damage. We hope to confirm or exclude these relationships in the final work on a larger group of patients.

The expression of the angiotensin-converting enzyme gene is regulated by microRNA-27a and microRNA-27b [39]. In a five-year longitudinal population-based study from Japan, circulating microRNA-27a was negatively associated with incident hypertension [40]. In a rat model, exercise increased microRNA-27a and -27b [41,42]. A loss of microRNA-27a induced cardiac dysfunction [43], thus suggesting a cardioprotective role of this molecule. Conversely, the authors from China revealed that the serum microRNA-27b levels were significantly higher in hypertensive patients with left ventricular hypertrophy (LVH) than in hypertensive patients without LVH and in healthy volunteers [44].

MicroRNA-21 is a molecule closely related to primary hypertension. By affecting SMAD7, vascular endothelial growth factor, renin pathways, and subclinical inflammation, microRNA-21 is involved in the development and progression of hypertension and HMOD, including left ventricular hypertrophy and arterial damage measured by the cIMT and PWV [45]. A post-exercise decrease in microRNA-21 expression was revealed in an animal model [46] and in women with breast cancer [47], which may be another mechanism for the positive effects of exercise on human health.

A meta-analysis of fourteen trials (15 interventions, 642 adult participants with arterial hypertension), involving five aerobic, two dynamic resistance, six combined, and two isometric resistance groups, showed that exercise interventions based on aerobic, combined, or isometric exercise were able to decrease the PWV [48]. Also, a meta-analysis of 26 studies in overweight and obese children revealed that aerobic exercise had a favorable effect on arterial stiffness, while the improvement was ineffective after resistance training and a combined training mode [49]. In our study, a single aerobic exercise session did not cause a significant change in any of the analyzed parameters of organ damage, including arterial stiffness. Nevertheless, we observed a substantially reduced total vascular resistance (TVR). According to Poiseuille’s law, the TVR correlates positively with the blood viscosity and vessel length but is inversely related to the fourth power of the radius. Thus, reductions in the TVR are strongly associated with changes in the vessel diameter; even small changes profoundly affect the vascular resistance. A larger lumen diameter and greater distensibility of blood vessels are structural adaptations to exercise that allow for lower peripheral resistance. We want to underline that our study is preliminary, and the analysis will be repeated on a larger group of hypertensive children and healthy peers.

Our study has several obvious limitations. First, the study sample is small and does not allow us to draw definitive conclusions. Second, there is no control group, which affects the analysis of both clinical data and microRNA expression analysis. Hence, we used the microRNA expression before exercise (in a given patient) instead of the expression in the control group as the baseline expression. The group is not ethnically and age-diverse. Thus, it is not easy to transfer these results to other populations. On the other hand, despite these limitations, the study’s strengths should be highlighted. First, it is the first pioneering analysis of microRNAs in children with hypertension. Second, in our study, we used (and described in detail in Section 2) an established method for assessing microRNA expression. All samples were analyzed by a single investigator (MS) previously trained by microRNA experts. Third, the patients’ vascular phenotype and blood pressure were assessed in extraordinary detail. An additional strength is the inclusion of a standardized intervention and the evaluation of virtually all parameters before and after exercise. We are aware of the limitations and are confident that the final study will answer the question of the role of microRNA in pediatric primary hypertension, HMOD, and its relationship to exercise in this group of patients.

## 5. Conclusions

To conclude, our preliminary study in 22 pediatric patients with primary hypertension revealed a single bout of standardized aerobic exercise-induced changes in microRNA expression together with a decrease in blood pressure, stroke volume, and total vascular resistance. We hypothesize that microRNA-133a might be a marker of left ventricular hypertrophy in children with primary hypertension. Exercise-induced alteration of microRNA-145 expression may cause a post-exercise reduction in the blood pressure and have a protective effect on arterial parameters in children with primary hypertension. Both microRNA-133a and microRNA-145 may induce epigenetic modifications affecting hypertension-mediated organ damage in children with primary hypertension, but this requires further studies.

## Figures and Tables

**Table 1:** Clinical and biochemical data of the study group.

Parameter	Value ± SD [Quartiles]
Number of patients (*n*)	22
Boys/girls (*n*, %)	18/4 (82%/18%)
Age [years]	15.1 ± 1.9 (from 11.3 to 18.0) [14.0–16.8]
Duration of gestation [weeks]	38.5 ± 2.2 [37–40]
Birth weight [g]	3343 ± 480 [3090–3600]
BMI Z-score	1.34 ± 0.93 [0.66–1.99]
Overweight patients (BMI 85–95 c) (*n*, %)	7 (32%)
Obese patients (BMI > 95 c) (*n*, %)	9 (41%)
GFR [mL/min/1.73 m^2^]	124.0 ± 11.7 [114–134]
Total cholesterol [mg/dL]	157.7 ± 40.2 [125–193]
LDL-cholesterol [mg/dL]	88.5 ± 31.6 [64–103]
HDL-cholesterol [mg/dL]	49.4 ± 13.4 [41–60]
Triglicerydes [mg/dL]	99 ± 40 [62–141]
Uric acid [mg/dL]	5.9 ± 1.1 [5.1–6.7]
Serum sodium [mmol/L]	140.0 ± 1.5 [139–141]
Serum potassium [mmol/L]	4.34 ± 0.22 [4.18–4.50]

*n*—number of patients, BMI—body mass index, GFR—glomerular filtration rate, LDL—low-density lipoprotein, HDL—high-density lipoprotein.

**Table 2:** Office and 24 h ambulatory blood pressure of the studied children.

Parameter	Before Exercise	After Exercise	*p*
SBP [mm Hg]	139.1 ± 12.0 [133–148]	134.9 ± 12.7 [125–145]	0.073
SBP Z-score	2.01 ± 0.92 [1.6–2.5]	1.66 ± 0.94 [0.9–2.5]	0.072
DBP [mm Hg]	82.2 ± 8.5 [75–88]	78.6 ± 8.8 [70–87]	0.010
DBP Z-score	2.3 ± 1.15 [1.5–3.0]	1.78 ± 1.14 [0.5–2.8]	0.010
24 h SBP [mm Hg]	133.1 ± 10.6 [124–141]	131.7 ± 9.9 [125–137]	0.296
24 h DBP [mm Hg]	75.0 ± 8.3 [68–82]	73.0 ± 7.4 [68–79]	0.019
24 h HR [beats/min]	74.2 ± 9.5 [68–80]	77.8 ± 10.3 [70–82]	0.004
24 h MAP [mm Hg]	101.5 ± 8.9 [94–109]	100.0 ± 7.8 [95–104]	0.133
24 h cSBP [mm Hg]	115.7 ± 10.2 [108–124]	114.3 ± 8.6 [109–118]	0.264
24 h cDBP [mm Hg]	77.5 ± 8.5 [69–86]	75.7 ± 7.7 [69–82]	0.049
SBP DIP [%]	10.5 ± 6.6 [5.3–13.6]	10.0 ± 7.2 [4.5–15.7]	0.712
DBP DIP [%]	14.0 ± 8.3 [6.6–18.3]	13.5 ± 8.5 [7.6–18.3]	0.755
24 h AIx 75 HR [%]	14.7 ± 6.58 [9–18]	15.7 ± 6.29 [11–18]	0.160
24 h TVR [dyn × s/cm^5^]	1557.8 ± 104.2 [1501–1619]	1509.4 ± 92.2 [1430–1579]	0.007
24 h PWV [m/s]	5.2 ± 0.3 [4.9–5.5]	5.2 ± 0.3 [5.0–5.3]	0.734
24 h stroke volume [mL]	74.5 ± 8.1 [71–79]	72.2 ± 8.4 [66–78]	0.011

SBP—systolic blood pressure, DBP—diastolic blood pressure, HR—heart rate, c—central blood pressure, AIx 75 HR—augmentation index normalized to the heart rate of 75 beats per minute, TVR—total vascular resistance, PWV—pulse wave velocity.

**Table 3:** Hypertension-mediated organ damage indicators in the studied children.

Parameter	Before Exercise	After Exercise	*p*
ACR [mg/g]	7.0 ± 6.7 [3.5–7.3]	8.4 ± 11.3 [3.6–6.8]	0.606
cfPWV [m/s]	5.2 ± 0.65 [4.8–5.6]	5.1 ± 0.75 [4.4–5.6]	0.255
cfPWV Z-score	−0.27 ± 0.9 [−0.9–0.3]	−0.48 ± 1.0 [−1.4–0.2]	0.234
cIMT [mm]	0.492 ± 0.05 [0.45–0.52]		NA
cIMT Z-score	2.0 ± 1.0 [1.4–2.8]		NA
LVM [g]	145.7 ± 44.5 [114.5–162.2]		NA
LVMI [g/m^2.7^]	32.8 ± 8.6 [26.8–37.6]		NA
LVMI [g/m^2^]	74.9 ± 20.3 [65.6–80.8]		NA

ACR—albumin-to-creatinine ratio; cfPWV—carotid–femoral pulse wave velocity; cIMT—common carotid artery intima–media thickness; LVM—left ventricular mass; LVMI—left ventricular mass index.

## Data Availability

Data used to support the findings of this study are included within the Supplementary Information files (Dataset.xlsx).

## Supplementary Materials

The following supporting information can be downloaded at https://www.mdpi.com/article/10.3390/jcm13226929/s1: Supplementary Table S1—microRNA names, miRbase accession name, and corresponding sequence.
