# Supplementary material for: MicroRNA-133a and MicroRNA-145 May Be Involved in the Development of Hypertension-Mediated Organ Damage in Children with Primary Hypertension—A Preliminary Study
Source: J Clin Med. 2024 Nov 18;13(22):6929. doi: 10.3390/jcm13226929 (PMC11595006; doi:10.3390/jcm13226929)
Supplement: Supplementary file 1 [file jcm-13-06929-s001.zip › Supplementary Table S1.pdf]

Supplementary Table S1 – microRNA names, miRbase accession name and corresponding sequence.

microRNA	miRBase accession	Sequence
cel-microRNA-39	MIMAT0000010	UCACCGGGUGUAAAUCAGCUUG
cel-microRNA-54	MIMAT0000025	UACCCGUAAUCUUCAUAAUCCGAG
microRNA-16-5p	MIMAT0000069	UAGCAGCACGUAAAUAUUGGCG
microRNA-21-5p	MIMAT0000076	UAGCUUAUCAGACUGAUGUUGA
microRNA-27a-3p	MIMAT0000084	UUCACAGUGGCUAAGUUCCGC
microRNA-27b-3p	MIMAT0000419	UUCACAGUGGCUAAGUUCUGC
microRNA-133a-3p	MIMAT0000427	UUUGGUCCCCUUCAACCAGCUG
microRNA-145-5p	MIMAT0000437	GUCCAGUUUUCCCAGGAAUCCCU
